# Flavonoids as inducers of white adipose tissue browning and thermogenesis: signalling pathways and molecular triggers

**DOI:** 10.1186/s12986-019-0370-7

**Published:** 2019-07-18

**Authors:** Xuejun Zhang, Xin Li, Huang Fang, Fengjin Guo, Feng Li, Anmin Chen, Shilong Huang

**Affiliations:** 1 Department of Orthopedics, First People’s Hospital of Yichang, No.4 Hudi Street, Yichang, 443000 Hubei Province China; 2 Department of Pediatrics, Wuhan Union Hospital, Tongji Medical College, Huazhong University of Science and Technology, No.1277 Jie Fang Avenue, Wuhan, 430022 Hubei Province China; 3 Department of Orthopedics, Tongji Hospital, Tongji Medical College, Huazhong University of Science and Technology, No.1095 Jie Fang Avenue, Wuhan, 430030 Hubei Province China

**Keywords:** Flavonoids, Brown adipose tissue, Browning, Obesity

## Abstract

**Background:**

Flavonoids are a class of plant and fungus secondary metabolites and are the most common group of polyphenolic compounds in the human diet. In recent studies, flavonoids have been shown to induce browning of white adipocytes, increase energy consumption, inhibit high-fat diet (HFD)-induced obesity and improve metabolic status. Promoting the activity of brown adipose tissue (BAT) and inducing white adipose tissue (WAT) browning are promising means to increase energy expenditure and improve glucose and lipid metabolism. This review summarizes recent advances in the knowledge of flavonoid compounds and their metabolites.

**Methods:**

We searched the following databases for all research related to flavonoids and WAT browning published through March 2019: PubMed, MEDLINE, EMBASE, and the Web of Science. All included studies are summarized and listed in Table 1.

**Result:**

We summarized the effects of flavonoids on fat metabolism and the specific underlying mechanisms in sub-categories. Flavonoids activated the sympathetic nervous system (SNS), promoted the release of adrenaline and thyroid hormones to increase thermogenesis and induced WAT browning through the AMPK-PGC-1α/Sirt1 and PPAR signalling pathways. Flavonoids may also promote brown preadipocyte differentiation, inhibit apoptosis and produce inflammatory factors in BAT.

**Conclusion:**

Flavonoids induced WAT browning and activated BAT to increase energy consumption and non-shivering thermogenesis, thus inhibiting weight gain and preventing metabolic diseases.

## Introduction

White fat cells are unilocular, and their main function is to store energy in the form of triglycerides. In contrast, brown adipocytes are multilocular, contain substantial numbers of mitochondria and have high expression uncoupling protein 1 (UCP1). Brown adipose tissue has been found in newborns and is involved in non-shivering thermogenesis. The primary function of brown fat is to transform energy into heat and maintain body temperature. BAT has long been thought to be absent in adult humans until Nedergaard [1] reported the discovery of some BAT in the supraclavicular and the neck regions of adult humans. In contrast to the components of classic BAT, Cannon and Nedergaard [2] found another kind of adipocyte in white adipose tissue after chronic treatment with the peroxisome proliferator-activated receptor (PPAR) γ agonist rosiglitazone; these other adipocytes are namely “Brite adipocytes” or “beige adipocytes” that also express UCP1 and proliferator-activated receptor-γ coactivator 1α (PGC-1α). These cells are also multilocular, with moderate mitochondrial content [3] and inducible expression of UCP1 and exhibit an interphase arrangement with white fat cells in WAT, thus are also called induced BAT (iBAT) [2]. Similar to BAT, iBAT also has thermogenic capacities [4] and the ability to prevent weight gain and metabolic disorders and promote whole-body energy balance [5, 6].

Although adults also hav brown fat, BAT metabolic effects and/or mass decline as healthy humans age [7, 8]. Ageing is associated with an increasing incidence of metabolic syndromes such as type 2 diabetes, obesity, non-alcoholic fatty liver disease (NFALD) and other disorders. The age-dependent disappearance of these brown adipocytes is associated with the development of insulin resistance and the accumulation of body fat [8]. Many studies have demonstrated that reversing age-related decreases in BAT or inducing WAT browning could be a potential strategy to treat age-related metabolic disorders [9–11]. However, in some hypermetabolic conditions (cancer, burns and massive trauma), studies have also found WAT browning and adipose tissue wasting. Researchers think that WAT browning enhances whole body energy expenditure causing a catabolic state of muscle protein breakdown and increased lipolysis, ultimately leading to cachexia [12].

**Fig. 1:**
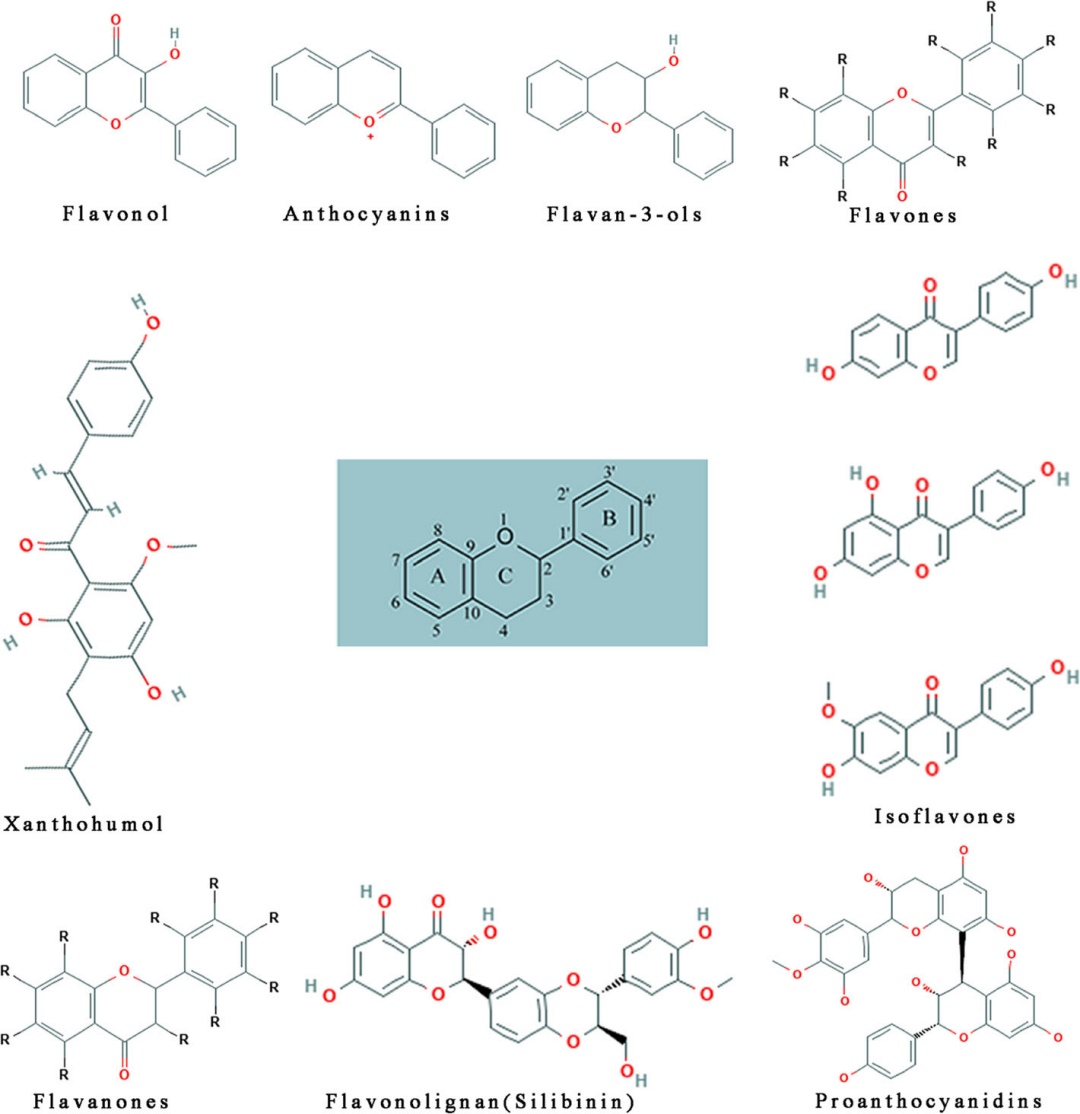
The basic structures of flavonoids, subgroups and derivatives

Flavonoids, members of the polyphenol family, are a large group of natural compounds with more than 4000 types and are mainly extract from fruits, vegetables, and teas [13]. According to their structure, flavonoids has been divided into 12 subgroups: anthoxanthins (flavone and flavonol), anthocyanidins, flavanones, flavanonols, flavans, and isoflavonoids. The basic structures of flavonoids are shown in Fig. 1. Six of flavonoids are found in significant quantities in our diet [13]. These active small compounds have been demonstrated to possess anti-inflammatory [14], antioxidative [15], anticancer [16, 17], anti-obesity activity, etc. [18]. In recent studies, several kinds of flavonoids have been found to induce WAT browning and promote energy balance in humans and animals [19–21]. Kang found that flavonoid derivatives increase energy expenditure through non-shivering thermogenesis [22]. Azhar identified some phytochemicals (guggulsterone, resveratrol, capsinoids etc) as inducers of browning in white adipose tissue [23]. Compared with flavonoids, non-flavonoids have a similar mechanism of promoting the browning of WAT. For example, resveratrol has been shown to induce the browning of WAT through the AMPK/PGC-1α/Sirt1 and PPARγ pathways. However, non-flavonoids also have a unique mechanism of promoting browning of WAT. For example, capsaicin and cinnamaldehyde combine with the intestinal transient receptor potential vanilloid 1 (TRPV1) receptor to activate the SNS, which in turn promotes WAT browning and thermogenesis. Considering the functional differences caused by the different structures of the different compounds, we chose to summarize the effects and mechanisms of flavonoids. However, none of the long-term follow-up clinical or in vivo studies have demonstrated that flavonoids can promote human health, leaving it impossible to say if these activities have any beneficial or detrimental effect on human health. In this review, we will summarize recent works on flavonoids and brown adipose tissue and discuss the mechanism underlying the promotion of WAT browning by flavonoids.

## Methods

We used the MeSH terms “flavonoid”, or “flavone” and “browning” or “brown adipose tissue” or “BAT” or “beiging” to search the following databases for all research related to flavonoids and WAT browning published through March 2019: PubMed, MEDLINE, EMBASE, and the Web of Science. Furthermore, we examined the reference lists of eligible articles and review studies by hand to identify additional studies. There were no restrictions with regard to species, age, sex, or publication type. The search was limited to articles published in English.

## Results

Studies were included when the following inclusion criteria were met: (1) the target compound of the study was flavonoids or their metabolites; (2) specific markers for brown fat were detected in the study; (3) the study researched related mechanisms; (4) the study indicated the concentration of related compounds and the processing time, and related results were reported; and (5) animal experiments were supported by the relevant ethics committees. Exclusion criteria included the following: (1) non-flavonoid-related research; (2) no mechanism was studied; (3) failure to pass ethical review; (4) no detection of brown-fat-related markers; (5) lack of reported dose, duration, or results. Unpublished studies and conference abstracts were also excluded because they cannot provide enough information. We classified all included studies by subgroup. All studies met the inclusion criteria are included in Table 1. We summarized the source of the compound, the animal species, age, cell lines or cell type, in vivo*/*in vitro study, dose/duration, effects and underlying mechanisms.

**Table 1:** Major subclasses of flavonoids with examples and studies on the effects of flavonoids on WAT browning

Compound	Source	In vivo*/*vitro	Dose/Duration	Effect	Ref.
Flavonol					
Quercetin	Onion-peel	C57BL/6 mice, 3 T3-L1 adipocytes	5 mg/g diet, 8 W 0-240 μM	AMPK/Sirt1/PGC-1α↑	[24]
Quercetin	Onion peel	HFD-induced metabolic Syndrome	0.8 mg/g diet	Nrf2/HO-1↑, NF-kB↓, attenuated oxidative stress and inflammation	[25]
Quercetin	Onion peel	HFD-fed mice	1 mg/g, 12 W	AMPK /Sirt1 pathway	[26]
Quercetin (Q) and Resveratrol (RSV)		HFD-induced obese Rats	15 + 30 mg/kg 6 W	perirenal WAT and interscapular BAT UCP-1↑	[27]
Quercetin	Onion peel	HFD-fed mice	1 mg/g, 12 W	sWAT browning and TG↓	[28]
Quercetin	Onion peel	3 T3-L1 adipocytes	10, 50, and 100 μM	AMPK pathway, ERK1/2 and JNK phosphorylation	[29]
Isorhamnetin	Metabolite of quercetin	3 T3-L1 adipocyte *ob/ob* mice	12.5 to 50 μM 10 mg/g, 4 W	PPARγ↓	[30]
Quercetin-rich onion peel extract	Onion peel	3 T3-L1 adipocyte	1to 50 μg/ml	PPARγ and C/EBPα↓, aP2 and LPL↓	[31]
Rutin	Mulberry	Db/Db and HFD-fed mice, C3H10T1/2 cells	1 mg/ml in water, 10 W10 μM	Sirt1/PGC-1α/Τfam↑	[19]
Rutin	Mulberry	HFD-induced obesity C57BL/6 mice	50 mg/kg, 8 W	PGC-1α↑	[32]
Anthocyanins					
Cy-3-G		db/db mice	1 mg/ml in water, 16 W	UCP1, Sirt1 and PGC-1α, PPARα	[33]
Cy-3-G	Mulberry	HFC-fed C57BL/6 J mice, C3H10T1/2 clone8 cells	200 mg/kg, 8 W 100, 200 μg/ml	PGC-1α, FGF21, eNAMPT	[34]
Cy-3-G		3 T3-L1 adipocytes	20–100 μM	AMPK, cAMP-C/EBPβ↑	[35]
Mulberry extract Cy-3-G	Mulberry	C3H10T1/2 MSCs	10 μg/ml	p38AMPK–PGC-1α-PRDM16	[36]
Flavan-3-ols					
Flavan-3-ol	Cocoa	ICR mice	10 mg/kg diet, 2 to 20 h	AMPK/ PGC-1α↑	[37]
Flavan-3-ol fraction	Cocoa	C57BL/J mice	50 mg/g, 2 W	PGC-1α/UCP-1↑MCAD	[38]
Flavan-3-ol fraction	Cocoa	HFD-fed Wistar rats	2 mg/g diet, 4 W	β-oxidation-related enzymes and UCP-1↑	[39]
Catechins	Tea	HFD-fed SD rats	5 mg/g diet, 5 W	BAT and UCP1↑	[40]
Theaflavins	Black tea	ICR mice	10 ml/kg, 2 to 20 h	AMPK/PGC-1α↑	[41]
Oolong,blackandpu-erh	Tea	ICR mice	2 g/100 ml drink for 7 days	AMPK/UCP-1↑, PPARγ and C/EBPα↓	[42]
Catechins	Green tea	HFD-fed SD rats,	100 mg/kg/d, 30 Days	PPARγ increased in sWAT and decreased in vWAT, PPARδ↑	[43]
Green tea extract	Green tea	SD rats	50 mg/kg, 24 h	NA-cAMP axis	[44]
(−)-epicatechin	Cacao	HFD-fed C57BL/6 mice, Human adipocytes	1 mg/g diet, 15days100nM	Mitochondrial biogenesis and fat browning	[45]
Flavones					
Chrysin	Flowers, honeycombs, and mushrooms	3 T3-L1 adipocytes	50 μM	AMPK/PGC-1α/UCP-1	[46]
Luteolin	Pepper, celery, thyme, peppermint	HFD-fed C57BL/6 mice	0.1 mg/g diet, 12 W	AMPK/PGC-1α signaling↑	[20]
Nobiletin	Citrus fruits	3 T3-L1 adipocytes and HIB1B brown adipocytes	100 μM	AMPK/PGC-1α and Sirt1	[47]
Sudachitin	Sudachi	C57BL/6 and db/db mice	5 mg/kg, 4 and 12 weeks	Sirt1–PGC-1α	[48]
Flavanones					
G-hesperidin	Peel of fruits	Wistar rats	60 mg/ml in water	BAT-SNA↑, CASNA↓	[49]
Naringenin		HFD-fed Ldlr(−/−) mice	10,30 mg/g, 4 W	PGC-1α/ PPARα	[50]
*Citrus aurantium* flavonoids	Hesperidin, Naringenin, and Nobiletin	3 T3-L1 adipocytes	0, 10, or 50 μg/ml	Akt/PPARγ and C/EBPα↓	[51]
Naringenin		Long-Evans hooded rats	0.03 to 0.12 mg/g, 6 W	PPARα↑	[52]
Isoflavones					
Isoflavones	Soy	Male SD rats	0.5 or 4 g/kg, 2 W	UCP, PPARα↑	[53]
Isoflavones	Soy food products	Long-Evans rats	600 mg/g diet, 33, 55 or 75 days of age	BNP Y↑, Thyroid↑, leptin and insulin↓	[54]
Genistein	Soyabeans	3 T3-L1 adipocytes	100 μM	C/EBPβ, PGC-1α, Sirt1↑	[55]
Daidzein	Soy isoflavones	HFD-fed Rats	50 mg/kg, 2 W	PPARγ and SCD1↓	[56]
Flavonolignan					
Silibinin	Milk thistle	Human ACS	10 μM	Sirt1, PPARα, PGC-1α↑	[21]
Proanthocyanidins					
Proanthocyanidins	Fruits, berries, beans, nuts, cocoa and wine	Wistar rats fed with a cafeteria diet	25 and 50 mg/kg diet, 4 months	Sirt1 and PGC-1α↑	[57]
Proanthocyanidin extracts	Chinese bayberry	HFD-fed obese SD rat	4, 26, 53%, 4 W	Sirt1, BMP4↑, C/EBP-α, PPAR-γ↓	[58]
Proanthocyanidin		Wistar rats	250 mg/kg	PGC-1α↑	[59]
Flavangenol	French martima pine bark	HFD-fed Wister rats	3 μg intraduodenal injection, 60 min	BAT-SNA↑	[60]
Procyanidin	Cacao liquor	HFD-fed C57BL/6 mice	5, 20 mg/g diet, 13 W	AMPKα/GLUT4/ PGC-1α↑	[61]
Xanthohumol					
Xanthohumol-rich hop extrac	*Humulus lupulus* L	3 T3-L1 adipocytes	10, 25 μg/ml	PPAR-γ, C/EBPα, aP2↓	[62]
Xanthohumol	Humulus lupulus L	3 T3-L1 adipocytes	0 to 100 μM	PPAR-γ, C/EBPα, aP2↓, apoptosis↑	[63]
Matured Hop Bittering Components		HFD-fed C57BL/6 J mice Wistar rats	0 to 2 mg/kg, 9 W 2 or 10 mg/kg, 90 min	BAT-SNA, PGC-1α, PRDM16, PPARγ↑	[64]
Plant Extract Mixture					
Black soybean seed coat extract	Black soybean	Male C57BL/6 mice	0 to2 mg/g diet 14 weeks	UCP1↑in BAT and WAT; TNF-α and MCP-1↓.	[65]
Extract of kumquat	Citrus fruits	HFD-fed C57BL/6 mice	10 mg/kg, 8 weeks, 3 months	PPARα↑	[66]
Puerariae flower extract	Kudzu flower	HFD-fed C57BL/6 J mice	50 mg/kg, 13.55 mg/kg ISOF, 6 W	UCP1↑in BAT	[67]
Olive Leaf Extract	Oleuropein	Human ASCs	0.27 and 0.37 mg/ml	Sirt1, PPARα, PGC-1α↑	[68]
E. cava polyphenol extract	Brown alga Ecklonia cava	HFD-fed C57BL/6 mice	100, 500 mg/kg/day 12 W	AMPK, PGC-1α, Sirt1↑	[69]

## Discussion

### Flavonoids and WAT browning

#### Flavonol

Flavonols are a group of 3-hydroxy-4-keto-flavonoids that mainly include kaempferols, quercetin, and rutin. Quercetin is extracted from onion peel and has been demonstrated to have many biological functions, such as antioxidant, anti-inflammatory and anti-obesity effects [70]. Many studies have demonstrated that quercetin supplementation prevents HFD-induced obesity and metabolic syndrome and increases the expression of UCP1 and thus thermogenesis through the adenosine monophosphate-activated protein kinase (AMPK) signalling pathway [24–27, 71, 72]. In diet-induced obese C57Bl/6 J mice, quercetin significantly increased the expression of UCP1 and Elovl3, specifically in subcutaneous white adipose tissue (sWAT). Quercetin also decreased plasma triglyceride (TG) levels and increased TG-derived FA uptake by sWAT as a consequence of WAT browning in HFD mice [28]. These results indicated that quercetin may induce WAT browning to achieve its anti-obesity effect through the AMPK-Sirt1 pathway [29, 73]. In 3 T3-L1 preadipocytes, quercetin and its metabolite isorhamnetin inhibited adipogenic differentiation by decreasing the expression of PPARγ, C/EBPα, FABP4, aP2, and lipoprotein lipase (LPL), but quercetin also increased the expression of brown-like adipocyte-specific genes, such as positive regulatory domain containing 16 (PRDM16), UCP1, fibroblast growth factor (FGF21), T-box transcription factor 1 (TBX1), PGC-1α and cell death-inducing DNA fragmentation factor alpha (DFFA)-like effector α (CIDEA) [30, 31, 74]. In conclusion, quercetin might prevent adipogenic differentiation but also induce the beiging of white adipocytes through the AMPK and PPARγ pathways to prevent obesity.

Rutin, also called vitamin P, is a kind of flavone glycoside. Rutin is found mainly in buckwheat and usually coexists with quercetin [75]. Rutin has been shown to protect mice from HFD-induced obesity and adipocyte hypertrophy, and to up-regulate the transcription of genes (deiodinase 2 (Dio2), Elovl3, PGC-1α, UCPs) involved in energy expenditure in BAT and to maintain glucose sensitivity [32]. In db/db and diet-induced obese (DIO) mice, rutin treatment significantly reduced adiposity, increased energy expenditure, and improved glucose homeostasis. The expression levels of UCP1, PGC-1α, PGC-1β, carnitine palmitoyltransferase 1 alpha (CPT-1α), and PPARα increased significantly in sWAT after rutin treatment. The underlying mechanism is as follows: rutin binds to and stabilize Sirt1 to increase Sirt1-mediated PGC-1α deacetylation, which stimulates Tfam transactivation and eventually augments the number of mitochondria and UCP1 activity in BAT [19]. Therefore, rutin has been thought to be of potential health benefits against diabetes and related disease [76].

Dihydromyricetin has been shown to stimulate irisin secretion partially through the PGC-1α pathway. Another study demonstrated that irisin could stimulate UCP1 expression in WAT and cause an increase in energy expenditure in mice [6, 77]. Our unpublished results indicated that dihydromyricetin also induced WAT browning. The underlying mechanism is that dihydromyricetin activates the PGC-1α/irisin axis.

#### Anthocyanins

Anthocyanins are a class of compounds including cyanidin, delphinidin, malvidin, pelargonidin, peonidin, and petunidin. Anthocyanins are mainly found in dark- coloured fruits and vegetables. Jin found that cyanidin-3-glucoside (Cy-3-G) treatment increased energy expenditure, reversed metabolic syndrome and enhanced BAT activity in obese db/db mice. Cy-3-G also induces brown-like (beige) adipocyte formation and increases UCP-1 expression and mitochondrial number and function in the sWAT of db/db mice [33]. In vivo, Cy-3-G significantly reduced the signs of metabolic syndrome and body weight induced by HFD [78, 79]. Another study found that Cy-3-G also reduced inflammatory cell infiltration in the heart and liver [80]. Yang found that in HFD-fed mice, Cy-3-G improved the function of BAT and regulated the expression of adipokines (NRG4 and PGC-1α) in BAT. In preadipocytes and C3H10T1/2 clone8 cells, Cy-3-G inhibited the release of FGF21 [34]. In vitro, a previous study showed that Cy-3-G promoted preadipocyte differentiation by elevating intracellular cyclic adenosine monophosphate (cAMP), promoting C/EBPβ expression, and increasing the expression of mitochondrial genes (TFAM, SOD2, UCPs), UCP1 protein and beige adipocyte markers (CITED1 and TBX1) in 3 T3-L1 cells [35]. Mulberry extract, which contains mainly Cy-3-G, was found to elevate the expression levels of UCP1, PGC-1α, and PRDM16 during brown adipogenesis through the p38 MAPK pathway in C3H10T1/2 mesenchymal stem cells [36]. Although we have enough animal data [34, 78–80] to prove the role of anthocyanins, we still lack a large amount of clinical data to prove that anthocyanins can reduce the risk of diseases in humans.

#### Flavan-3-ols

Flavan-3-ols are a group of flavonoid substances including catechin, epicatechin gallate, epigallocatechin, proanthocyanidins, theaflavins, and thearubigins that are found in some plant foods such as cocoa beans, wine, and certain fruits. Flavan-3-ols consist of monomers and oligomers composed of catechins and their derivatives. In an in vivo study, the mRNA levels of UCP1 and PGC-1α in BAT increased significantly 2 h after the administration of flavan-3-ols, and the serum adrenaline concentration was significantly increased 2 h after treatment with flavan-3-ols [37]. In another study, after 2 weeks of administration of flavan-3-ols, the levels of UCP1 and mitochondria also increased significantly in BAT, which indicated that flavan-3-ols enhanced lipolysis and promoted mitochondrial biogenesis [38]. When HFD-fed rats were treated with flavan-3-ols, the expression levels of UCP1 protein increased in the BAT group compared with the levels in the HFD group [39].

The monomers of flavan-3-ols include catechin, (+)-catechin, (−)-epicatechin, (−)-epigallocatechin, (+)-gallocatechin, and their gallate derivatives. Catechins and their derivatives are abundant in tea and can promote energy balance. In an in vivo study, catechin was shown to reduce perirenal WAT weight and increase UCP1 mRNA expression in BAT after the consumption of a normal-fat diet for 8 weeks. The researchers concluded that catechins suppressed body fat accumulation by increasing UCP1 expression in BAT [40]. In another study, a single oral treatment with theaflavins significantly increased REE and the UCP1 and PGC-1α levels in BAT after 2 h. The researchers believed that theaflavins enhanced systemic energy expenditure by promoting AMPK1α phosphorylation and UCP1 and PGC-1αexpression in BAT [41]. Catechins and their derivatives are the main flavonoid components in tea. Several studies have reported that the intake of tea enhanced the phosphorylation of AMPK and modulated the PPAR pathway to increase the expression of UCP1 in WAT and thermogenesis through sympathetic stimulation [42–44]. (−)-Epicatechin (Epi), a cacao flavanol, increased fatty acid metabolism and upregulated the expression of brown adipose tissue-specific proteins in a high-fat diet-induced mouse model of obesity and cultured human adipocytes [45].

#### Flavones

Flavones are another subclass of flavonoids and include chrysin, luteolin, apigenin, and baicalein. Chrysin is a natural flavone found in flowers, honeycombs, and mushrooms. Choi found that chrysin significantly enhanced the expression levels of brown fat-specific genes (CIDEA, PGC-1α, PRDM16, TBX1, TMEM26, and UCP1) and the protein levels of brown fat markers, including CEBP/β, PGC-1α, PRDM16 and UCP1 in 3 T3-L1 adipocytes, suggesting possible conversion of white adipocytes into beige cells. In another study, they found that chrysin promoted the BAT phenotype through an AMPK-mediated pathway. The AMPK pathway inhibitor dorsomorphin reduced the expression of UCP1, PRDM16, and PGC-1α while the activator AICAR elevated the expression of these brown fat-specific genes [46]. Luteolin is a natural flavonoid and is most often found in leaves of pepper, celery, thyme, peppermint, and honeysuckle. Zhang reported that dietary luteolin supplementation increased energy expenditure in both HFD-fed and LFD-fed mice through the upregulation of thermogenic genes in brown and subcutaneous adipose tissues. Luteolin promotes the differentiation of subcutaneous adipose cells into brown fat cells, and it works by promoting adipocyte differentiation through the activation of the AMPK/PGC-1α pathway [20]. Nobiletin (NOB) is a polymethoxylated flavone isolated from citrus peels. Yun found that NOB not only activated HIB1B brown adipocytes but also induced mitochondrial biogenesis and browning of 3 T3-L1 white adipocytes. NOB also ameliorates stress and inhibits autophagy in adipocytes to sustain the brown adipocyte-like phenotype. The researchers found that NOB induced PKA and activated AMPK and consequently increased the expression of PGC-1α and UCP1. Inhibiting PKA and p-AMPK by H-89 and dorsomorphin abolished the expression of PGC-1α and UCP1 [47]. Sudachitin is a polymethoxylated flavone that is found in *Citrus sudachi hort*. ex. Shirai. In HFD-fed mice, sudachitin treatment resulted in lower body weight, improved glucose tolerance, and better insulin sensitivity. Moreover, the mRNA transcripts of UCP1 and UCP3 were significantly increased in WAT and adipocyte size and number also decreased after 12 weeks of sudachitin treatment [48].

#### Flavanones

Flavanones include hesperetin, naringenin, eriodictyol, etc. Flavanones are found in the peels of fruits such as satsuma mandarin orange and Valencia orange and are often found in plants as glycosides. One of the flavanones, hesperetin, has been found to increase thermogenesis. Researchers have found that oral administration of 60 mg of G-hesperidin increased interscapular BAT-SNA but decreased cutaneous sympathetic nerve activity (CASNA) in rats, and significantly increased subcutaneous body temperature (BT) [49].

Naringenin, a flavonoid found in a variety of fruits and herbs, has also been considered to be a bioactive compound that can protect against adiposity. A large amount of evidence has indicated that naringenin prevents metabolic syndrome by inhibiting diet-induced dyslipidaemia [50], lipogenesis [81] and adipogenesis [51]. Furthermore, naringenin supplementation activates PPARα and upregulates fatty acid oxidation target genes [52]. Naringenin increases hepatic fatty acid oxidation through the PGC-1α/ PPARα-mediated pathway [50]. In an unpublished study, the author showed that naringenin increased the expression of UCP1 and Sirt1 in primary human omental adipocytes in a dose-dependent manner [23]. In a soy protein diet-fed SD rat model, the author found that the protein, not the isoflavones, reduced hepatic lipogenesis, but they also found that the isoflavones regulated hepatic fatty acid oxidation and upregulated the expression of UCPs in BAT through a PPARγ-dependent mechanism [53].

#### Isoflavones

Isoflavones are mainly found in the Fabaceae family. Isoflavones include daidzein, genistein, glycitein, biochanin A, formononetin, and their metabolites. Lephart found that diets rich in isoflavones increased T3 levels and UCP1 mRNA levels in the BAT of Long-Evans rats, but the core body temperature decreased except near the end of the dark phase of the dark/light cycle [54]. Genistein is found in particularly high levels in soybean. Aziz found that genistein treatment changed the lipid distribution of 3 T3-L1 adipocytes, reduced white adipocyte-specific genes and increased brown/beige adipocytes specific genes. They also found that genistein stimulated WAT browning by activating Sirt1 to promote the expression of UCP1, C/EBPβ, and PGC-1α. They concluded that genistein acts directly on adipocytes or on adipocyte progenitor cells to programming the cells metabolically to adopt features of beige adipocytes [55]. Another kind of isoflavone, daidzein, was also found to prevent diet-induced obesity. Chronic treatment with daidzein for 14 days, reduced weight gain and fat content in the liver. This general physiological effect shows a complex interaction of many different factors through various possibly interrelated pathways and with a particular role of the inhibition of lipogenesis, involving PPARγ and the enzyme SCD1 [56].

#### Flavonolignan

Silibinin belongs to the flavonolignangroup and is the major active constituent found in milk thistle (*Silybum marianum*). Volti found that Silibinin treatment affects the adipogenic differentiation and lipids of mature adipocytes of human adipose tissue-derived mesenchymal stem cells (ASCs). In their study, silibinin was added either at the early or late stage of adipogenic differentiation, Silibinin both increased BAT-specific gene expression (Sirt-1, PPARα, PGC-1α, and UCPs) and also decreased WAT specific gene expression (PPARγ, fatty acid-binding protein 4 (FABP4)). Moreover, when mature adipocytes formed, silibinin treatment decreased the lipid droplets in mature adipocytes. This result indicates that silibinin induces thermogenesis and WAT browning by stimulating Sirt1, PPARα, and PGC-1α [21].

#### Proanthocyanidins

Proanthocyanidins are oligomeric flavonoids, mainly found in fresh grapes, red wine, and other dark pigmented fruits. In a rat model of HFD-induced obesity, propanthocyanidin supplementation inhibited the weight gain induced by a high-fat diet, increased the activity of cytochrome c oxidase activity, and enhanced UCP1 expression in brown adipocytes. The data indicate that chronic administration of proanthocyanidins enhances thermogenic capacity and improves mitochondrial function in the BAT of cafeteria-diet-induced obese rats [57]. Zhou found that proanthocyanidin extracts (PEs) from Chinese bayberry play an anti-obesity role by upregulating the expression of Sirt1, thus inducing the deacetylation of PPARγ, downregulating the expression of C/EBP-α and upregulating the expression of BMP4 to induced white-to-brown adipocyte transdifferentiation [58]. In another study, acute administration of Proanthocyanidin extract significantly improved lipid metabolism, and increased energy metabolism-related genes such as PGC-1α, and upregulated the oxidative capacity of skeletal muscle and BAT mitochondria [59]. Flavangenol is mainly found in French maritime pine bark. HFD-induced obesity was suppressed by flavangenol ingestion, and acute intraduodenal (ID) injection of flavangenol elevated BAT-SNA and inhibited gastric vagal nerve activity (GVNA) in anaesthetized rats. In addition, flavangenol elevated BAT-temperature in conscious rats. These results indicate that flavangenol inhibits obesity by influencing autonomic nerves and the thermogenic response [60].

#### Xanthohumol

Xanthohumol is a prenylated flavonoid found in the female inflorescences of *Humulus lupulus*. In an in vitro study, xanthohumol was demonstrated to inhibit preadipocyte differentiation and intracellular fat droplet accumulation [62] and induce apoptosis through oxidative stress in mature adipocytes [63]. In vivo, xanthohumol also inhibits HFD-induced weight gain and promotes lipid metabolism [82]. Xanthohumol also increases the energy expenditure of white and brown preadipocytes, hepatocytes and myocytes [83]. This effect was mediated by increasing the production of ROS, which leads to the activation of AMPK and PGC-1α and increasing uncoupling and oxygen consumption [83]. The administration of mature hop plants to rats induced thermogenesis and UCP1 expression in BAT. The authors found that the administration of mature hop plant components increased the cAMP concentration in BAT and activated the β-adrenergic signalling cascade, thereby modulating sympathetic nerve activity. They concluded that BAT-SNA activation plays an important role in mature hop component-induced thermogenesis [64]. Because xanthohumol can increase the oxygen consumption rate and the potential for chemical uncoupling, it is thought that xanthohumol may induce this metabolic change through systemic thyroid hormone signalling. In a xanthohumol-feeding rat experiment, xanthohumol affected tetraiodothyronine (T4) binding and distribution both in vivo and in vitro. Xanthohumol also moderately increased serum thyroid stimulating hormone (TSH) and triiodothyronine (T3) levels [84]. Additionally, other groups found acute administration of xanthohumol to increased iodide uptake after 3 days in nontransformed rat thyrocytes [85]. Xanthohumol might impact BAT activity through thyroid hormone signalling.

#### Plant extract mixture

Black soybean seed coat extract (BE) is a polyphenol-rich food material that mainlyconsists of Cy-3-G, catechins, and procyanidins. In HFD-fed C57BL/6 mice, BE exerted a protective effect against body weight gain and rescued glucose metabolism; BE also increased UCP1 expression in BAT. Researchers concluded that dietary BE consumption enhanced energy expenditure by upregulating UCPs expression [65]. *Fortunella margarita* fruit extract (FME) mainly contains polyphenols and flavonoids including neoeriocitrin and poncirin. The administration of FME along with an HFD blocked the HFD-induced body weight gain and decreased serum lipid levels. Consumption of the FME diet also increased the expression of UCP-2 but not UCP1 in BAT, and the expression of PPARα and its target genes in the liver increased significantly [66]. Cacao liquor procyanidin (CLPr) extract, mainly consists of catechin, epicatechin, and procyanidins. CLPr suppressed HFD-induced metabolic disorder in WAT. CLPr also promoted the translocation of glucose transporter 4 (GLUT4) and the phosphorylation of AMPKα in the plasma membrane of skeletal muscle and BAT. Phosphorylation of AMPKα was also enhanced in the liver and WAT. CLPr upregulated the gene and protein expression levels of UCP1 in BAT and UCP-3 in skeletal muscle [61]. *Puerariae* flower extract mainly consists of isoflavones. These compounds may increase energy expenditure by upregulating BAT UCP1 expression in HFD-fed C57BL/6 J male mice [67]. Olive leaf extract (OLE) contains a wide variety of phenolic acids, phenolic alcohols, flavonoids, and secoiridoids. The major component of these compounds is oleuropein and its major metabolite hydroxytyrosol. Researchers showed that OLE treatment induces thermogenesis by activating of UCP1, Sirt1, PPARα, and PGC-1α. OLE significantly decreases the expression of genes involved in adipogenesis and upregulates the expression of mediators involved in thermogenesis and lipid metabolism. OLE treatment resulted in a significant increase in pAMPK and HO-1 expression during adipose differentiation [68]. Green tea extracts (GTEs), particularly the catechins and epigallocatechin gallate (EGCG), reduce the expression of Ap2 in BAT, increase the expression of PGC-1α and vascular endothelial growth factor α (VEGFα), counteract the whitening of the BAT and induce the browning process in the WAT of HFD-induced obese mice [86]. Brown alga ecklonia cava polyphenol extract has been demonstrated to reduce HFD-induced obesity and metabolic syndrome and might have potential anti-obesity effects via the regulation of hepatic lipid metabolism, inflammation, and oxidative stress through the activation of AMPK/Sirt1 and the regulation of its downstream genes in HFD-induced obese mice [69].

### The pathways involved in flavonoid-induced WAT Browning

#### Sympathetic nervous system

The sympathetic nervous system (SNS) plays a decisive role in the thermogenesis of brown adipose tissue. When the body is exposed to cold, the temperature-sensitive neurons located on the skin surface feel cold stimulation, activate the sympathetic nervous system and release adrenaline, which binds to its receptors, promotes the activation of brown adipocytes and the browning of white adipose tissue and increases the expression of UCP-1 to generate heat [87]. Cold exposure increases Sirt1 phosphorylation/activity in both skeletal muscle and BAT, increasing thermogenesis and insulin sensitivity through the deacetylation of PGC-1α and other protein targets. Sirt1 increases insulin sensitivity and glucose control in skeletal muscles, triggers the browning of white fat and increases BAT activity. Adrenergic stimulation of BAT increases intracellular cAMP release and activates protein kinase A (PKA), leading to p38 MAP kinase activition and phosphorylation of nuclear-thermogenic-related genes such as ATF2 and PGC-1α to increase the transcription of the UCP1 gene [88]. The activation of PKA also boosts lipolysis in BAT cells [89]. AR-β activation of p38 MAP kinase in brown adipocytes activates the transcription of UCP1 and PGC-1α genes for adaptive thermogenesis, mitochondrial biogenesis and fatty acid oxidation. β3 adrenoceptor (β3-AR) stimulation leads to PGC-1α induction, which drives PPAR activation and mitochondrial biogenesis. The amount of heat produced by BAT mainly depends on the degree of activation of BAT sympathetic nerves, the extent of the subsequent norepinephrine release, and the intensity of the binding of released norepinephrine to the adrenergic receptors. In a case report study, a man with pheochromocytoma exhibited elevated plasma catecholamine and urinary catecholamine metabolite levels; PET/CT revealed increasing BAT activity in the neck, supraclavicular, axillary, mediastinal, paravertebral, and perinephric regions, which disappeared after resection of the tumour [90]. In contrast, blocking adrenergic receptors with propranolol completely diminished FDG uptake in BAT areas, suggesting the involvement of these adrenergic receptors in BAT activation in humans [91]. The catechins in green tea are believed to influence energy expenditure through the inhibition of the enzyme catechol-0-methyl transferase [92–94]. This enzyme is responsible for the degradation of catecholamines including norepinephrine. Because the degradation of norepinephrine and epinephrine are slowed, continuous stimulation of adrenergic receptors occurs with a resultant increase in energy expenditure and fat oxidation.

#### Thyroid hormone

Thyroid hormones (THs) are important physiological modulators of lipid metabolism. Brown adipose tissue is the main target tissue of THs. In brown adipocytes, THs can enhance thermogenesis by promoting the expression of UCP-1 in mitochondria. This is achieved by thyroid hormone receptors (TRs) interacting with PGC-1α and binging to the UCP1 enhancer region, resulting in increased UCP1 expression in mitochondria [95]. Total TRβ knockout mice present with defective adaptive thermogenesis and reduced BAT UCP1 expression, whereas the selective TRβ agonist GC1 along with noradrenaline increases UCP1 expression in brown adipocytes [96, 97]. Several lines of evidence indicate that THs regulates WAT browning. Medina-Gomez found that low doses of the T3 metabolite triiodothyracetic acid (TRIAC) induces ectopic expression of UCP1 in abdominal WAT [98]. López et al. found that intracerebroventricular (i.c.v.) administration of THs decreased the activity of hypothalamic AMPK but increased BAT sympathetic nerve activity and UCP1 expression, which was associated with weight loss without affecting food intake [99]. The researchers also found that inhibition of thyroid hormone receptors in the ventromedial hypothalamus (VMH) reverses the weight loss associated with hyperthyroidism. They concluded that THs activates TRs in the VMH to regulate BAT function through the SNS. Importantly, the effects of T3 on energy expenditure, thermogenesis and body weight were abolished in UCP1 knockout mice [100]. In human adipose tissue-derived multipotent cells, T3 treatment induced PGC1α and UCP-1 expression and mitochondrial biogenesis in a TRβ-dependent manner [101]. In a thyroid cancer patient case study, ^[18F]^FDG-PET/CT scanning revealed that T4 supplementation for 14 days increased radioactive glucose uptake and UCP-1 expression in suprascapular BAT and subcutaneous WAT regions [102]. Recent human data demonstrated that UCP1 expression in WAT is associated with serum T4 levels, suggesting that THs is positively associated with fat browning [103]. Lephart found that after feeding Long-Evans rats with a diet low in isoflavones, body and adipose tissue weights decreased but circulating T3 levels increased while body temperatures decreased with soy consumption. They thought the results were related to isoflavones mimicking the oestrogen effect to increase T3 and T4 [54]. In an in vitro study, kaempferol (KPF) increased energy expenditure and modified metabolic gene expression (UCP-3, PGC-1α) by activating the cAMP-PKA pathway. This result may be due to kaempferol increasing Dio2 activity by regulating T3 expression. The effect of KPF can be mimicked by dibutyryl cAMP, a stable cAMP analogue [104]. The synthetic flavonoid EMD 21388 has been demonstrated to inhibit T4 production and increase T3 production [105]. In conclusion, flavonoid consumption might increase T3 to induce WAT browning and upregulate UCP-1 expression in BAT. Central T3 regulates hepatic metabolism through the vagus nerve and BAT through the SNS, leading to increased lipid oxidation and thermogenesis. This physiological pathway is mediated by AMPK (specifically in SF1 neurons of the VMH), which also exerts a dichotomic action on ceramide-induced ER stress and C-Jun amino-terminal kinase (JNK1).

#### AMPK-PGC-1α/Sirt1 signalling pathway

AMPK is an enzyme (EC 2.7.11.31) that is highly expressed in the brain, liver, skeletal muscle and BAT, and plays a role in energy metabolism and regulating thermogenesis [106]. AMPK activates glucose and fatty acid uptake and oxidation when cellular energy is low. The enzyme complex comprises three subunits: a catalytic α subunit and two regulatory subunits, β and γ. The catalytic α subunit is mainly found in rodents, and the α1 isoform is predominant in the brain and WAT whereas α2 is mainly expressed in muscle. In C57Bl/6 mice, AMPKα1 is the dominant isoform that is mainly expressed in BAT [107]. Chronic cold exposure selectively stimulated the AMPK*α*1 isoform and maintained the high mitochondrial density in BAT. Recent data demonstrated that the activation of AMPK leads to PGC1α-mediated mitochondrial biogenesis and UCP-1 expression in BAT. PGC-1α is an important regulatory factor in the process of mitochondrial formation, oxidative metabolism and thermogenesis in BAT. PGC-1α is a master regulator of BAT thermogenesis [108]. PGC-1α coactivates various nuclear receptors for the transcriptional induction of UCP1 and other mitochondrial genes involved in mitochondrial biogenesis and oxidative metabolism [109]. AMPK and Sirt1, two key regulators of energy metabolism, can increase PGC-1α expression and phosphorylation [110, 111]. Moreover, AMPK can also enhance Sirt1 activity by increasing cellular nicotinamide adenine dinucleotide (NAD+) levels to induce PGC-1α deacetylation and activation [73, 112]. AMPK/PGC-1α signalling dominantly regulates differentiation and function in brown and beige fat [106, 112, 113]. Recent studies have shown that flavonoids can promote WAT browning and thermogenesis and inhibit adipocyte differentiation through the AMPK/PGC-1α pathway. For instance, luteolin enhanced energy expenditure and upregulated thermogenic genes in brown and subcutaneous adipose tissues (SAT). Luteolin has also been demonstrated to suppress adipogenic differentiation by activating AMPK/Sirt1 signalling. Luteolin treatment elevated the expression of UCP-1 and the activity of AMPK/PGC-1α signalling molecules in differentiated primary brown and subcutaneous adipocytes, which were fully mimicked by the AMPK activator 5-amino-4-formamide imidazolium ribonucleotide (AICAR). Furthermore, the AMPK inhibitor compound C could reverse the effects of luteolin and AICAR [20]. These results indicate that luteolin induces adipocyte browning and thermogenesis by activating AMPK/PGC-1α signalling. Other flavonoids, such as rutin [19], Cy-3-G [36], and chrysin [46], have also been demonstrated to induce adipocyte browning and thermogenesis by activating AMPK/PGC-1α signalling.

#### PPARs

Peroxisome proliferator-activated receptors (PPARs) are a group of nuclear transcription factors that function by regulating cellular differentiation, development, and energy metabolism and tumourigenesis. There are three types of PPARs: PPARα, β or δ, and γ. PPARα is mainly expressed in the liver, kidney, heart, muscle and adipose tissue and mediates the hypotriglyceridaemic effect of fibrates by inducing mitochondrial and peroxisomal-oxidation by decreasing the plasma concentration of triacylglycerol-rich lipoproteins [114]. PPARδ is mainly expressed in the brain, BAT, and skin. PPARδ is one of the central regulators of adipogenesis that promotes lipid storage in adipocytes [113]. PPARδ regulates the expression of genes required for fatty acid oxidation and energy dissipation, which led to improved lipid profiles and reduced adiposity [115]. PPARγ is mainly expressed in WAT, internal organs and macrophages. In mature adipocytes, PPARγ regulates the expression of genes involved in free fatty acid uptake and triglyceride synthesis, thereby increasing the ability of WAT to store triglycerides [116]. The PPARγ agonist thiazolidinedione can also induce a brown-like phenotype in white adipocytes by promoting the expression of brown adipocyte-specific genes and inhibiting visceral WAT genes [117]. The mechanism of this “browning” effect is related to the Sirt1-dependent PPARγ deacetylation of Lys268 and Lys293, which is required to recruit the BAT programme coactivator PRDM16 to PPARγ, leading to the selective induction of BAT genes and the repression of visceral WAT genes associated with insulin resistance. [118]. Increasing the levels of PPARδ in WAT is suggested as a potential strategy to treat obesity [119]. In a diet-induced obesity model, the PPARα agonist fenofibrate, elicits weight loss and increases β3-AR, PGC-1α and UCP-1 in brown adipocytes [120]. YAN et al. found that green tea catechins increased PPARδ but not PPARα levels in both BAT and WAT. In addition, the expression levels of PPARδ down-stream genes such as alternative oxidase (AOX), CPT1, and UCP1 were increased [43]. Once again, PPARα controls the transcription of this essential gene, which interacts with PGC-1α to provide the machinery necessary for the transdifferentiation or differentiation of the *brite* adipocyte [121].

## Conclusion

**Fig. 2:**
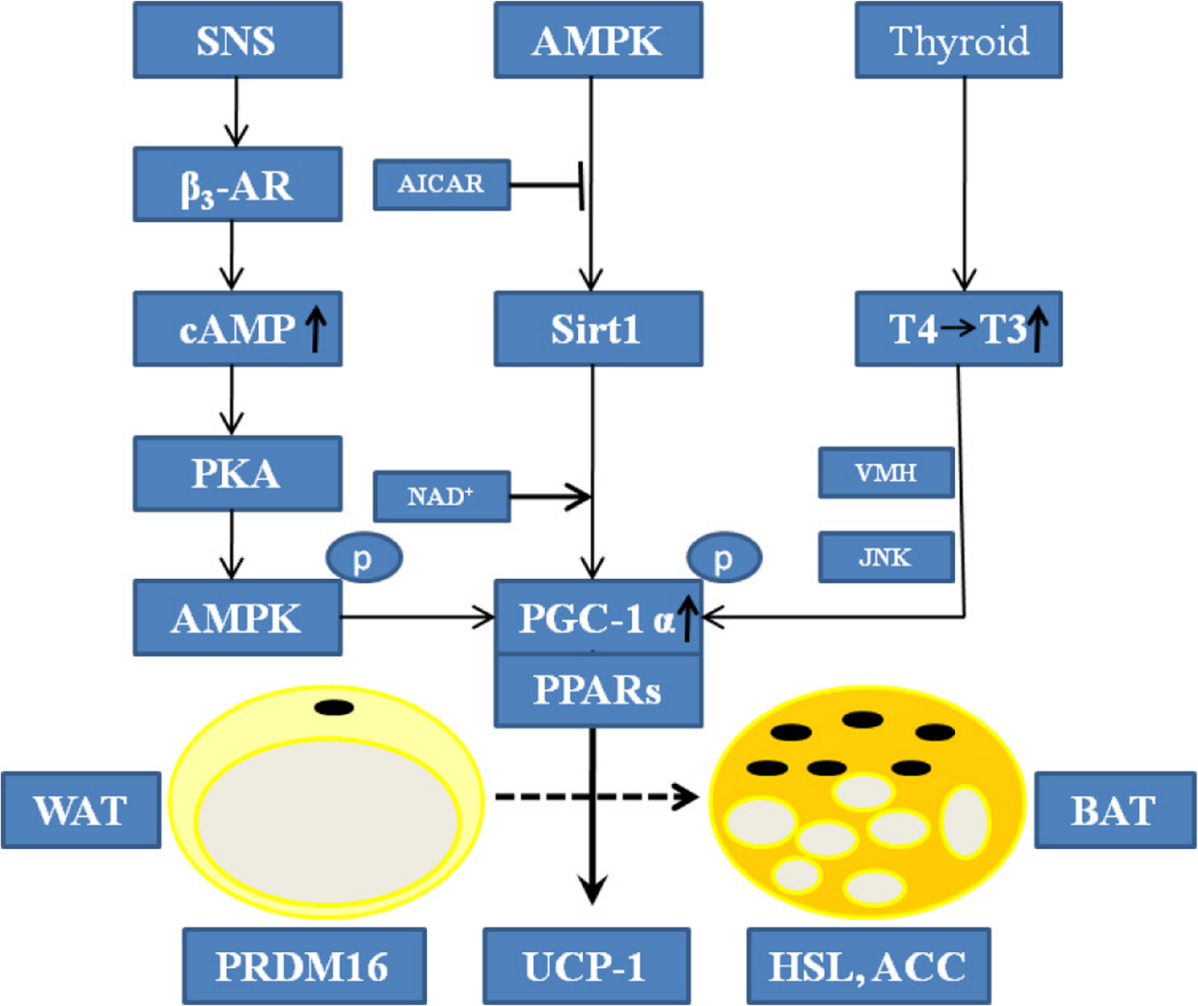
The signaling pathways and mechanisms whereby flavonoids promote WAT browning. SNS: sympathetic nervous system; β3-AR: β3 adrenocepter; cAMP: cyclic adenosine monophosphate; PKA: protein kinase A; AMPK: adenosine monophosphate-activated protein kinase; AICAR: 5-Amino-4-formamide imidazolium ribonucleotide; Sirt1: silent mating type information regulation 2 homolog 1; VMH: ventromedial hypothalamus; JNK: C-Jun amino-terminal kinase; T4: tetraiodothyronine; T3: triiodothyronine; NAD+: nicotinamide adenine dinucleotide; PGC-1α: proliferator-activated receptor-γ coactivator 1α; PPAR: peroxisome proliferator activated receptor; WAT: white adipose tissue; BAT: brown adipose tissue; PRDM16: positive regulatory domain containing 16; UCP-1: uncoupling protein 1; HSL: hormone sensitive lipase; ACC: acetyl-coenzyme A carboxylase

In this study, we summarized the role of flavonoids in metabolic diseases, and analysed the specific mechanism of flavonoid-induced WAT browning (Fig. 2). Flavonoids activated the SNS and promoted the release of adrenaline and thyroid hormones to increase thermogenesis and induce WAT browning through the AMPK-PGC-1α/Sirt1 and PPAR signalling pathways. This will help us better understand the benefits of flavonoids and their mechanism. Despite our positive results in animal experiments, there is still a lack of clinical trials to confirm the efficiency and safety of flavonoids in the human body. Mark found that inflammation reduces the expression of UCP-1 in mature brown adipocytes but that resveratrol partly reduced the downregulation of UCP-1 induced by IL1β [122]. Pro-inflammatory factor-induced apoptosis plays an important role in the acquisition of terminally differentiated phenotype of brown adipocytes [123]. Flavonoids may promote brown preadipocyte differentiation, inhibit apoptosis and produce inflammatory factors in BAT. Flavonoids elevate energy expenditure by activating the sympathetic nervous system and increasing UCP-1 mRNA in BAT and plasma catecholamine [124].

However, some unknown aspects and limitations remain to explored: (1) After intestinal absorption, flavonoids are metabolized in the intestinal and hepatic cells and appear as metabolites in the urine and blood [125]. In humans, the peak plasma concentrations of flavonoids absorbed and metabolized into the blood and urine are low. However, the roles of their metabolites may be different from parent compounds [126]. A potential need, therefore, is to precisely determine the lowest effective concentration of flavonoids. Another concern is whether this minimal effective concentration is obtainable after intestinal absorption and metabolism. Likewise, an important dogma would be the relative contributions of parent flavonoids and their metabolites to biological responses under considerations. (2) Additionally, the bioavailability of flavonoids is low due to limited absorption, extensive metabolism, and rapid excretion. However luckily; to date, no adverse effects have been found due to the high dietary intake of flavonoids from plant-based food in healthy people. Under special circumstances, however, like (cancer, burns and massive trauma), the benefits of promoting WAT browning by flavonoids must be weighed versus some reported adverse effects in these conditions. Further clinical trials are warranted to delineate their exact roles, safety and mechanisms. (3) Besides, some flavonoids are known to be phytoestrogens. Accordingly, although some studies found that flavonoids influence sex-hormone-dependent signaling pathways and protect against breast and prostate cancers, it is crucial to probe also whether and how they may also interfere with the synthesis and activity of such endogenous hormones [127].

## Data Availability

Not applicable.

## Abbreviations

ACC: Acetyl-coenzyme A carboxylase
AICAR: 5-Amino-4-formamide imidazolium ribonucleotide
AMPK: Adenosine monophosphate-activated protein kinase
AOX: Alternative oxidase
ASCs: Human adipose tissue derived mesenchymal stem cells
ATP: Adenosine triphosphate
BAT: Brown adipose tissue
BE: Black soybean seed coat extract
BT: Body temperature
C/EBP: CCAAT/enhancer-binding protein beta
cAMP: Cyclic adenosine monophosphate
CASNA: Cutaneous sympathetic nerve activity
CIDEA: Cell death-inducing DNA fragmentation factor alpha (DFFA)-like effector α
CLPr: Cacao liquor procyanidin
CPT-1α: Carnitine palmitoyltransferase 1 alpha
Cy-3-G: Cyanidin-3-glucoside
EGCG: Epigallocatechin gallate
FABP4: Fatty acid-binding protein 4
FME: *Fortunella margarita* fruit extract
GLUT4: Translocation of glucose transporter 4
GTEs: Green tea extracts
GVNA: Gastric vagal never activity
HFD: High-fat diet
HSL: Hormone sensitive lipase
JNK: C-Jun amino-terminal kinase
KPF: Kaempferol
LPL: Lipoprotein lipase
NAD +: Nicotinamide adenine dinucleotide
NOB: Nobiletin
OLE: Olive leaf extract
p38 MAPK: p38 mitogenactivated protein kinase
PE: Proanthocyanidin extracts
PGC-1α: Proliferator-activated receptor-γ coactivator 1α
PKA: Protein kinase A
PPAR: Peroxisome proliferator activated receptor
PRDM16: Positive regulatory domain containing 16
Sirt1: Silent mating type information regulation 2 homolog 1
SNS: Sympathetic nervous system
sWAT: Subcutaneous white adipose tissue
T3: Triiodothyronine
T4: Tetraiodothyronine
TBX1: T-box transcription factor 1
TG: Triglyceride
TH: Thyroid hormones
TRIAC: T3 metabolite triiodothyracetic acid
TRPV1: Transient receptor potential vanilloid 1
TRs: Thyroid hormone receptors
TSH: Thyroid stimulating hormone
UCP-1: Uncoupling protein 1
VEGF: Vascular endothelial growth factor
VMH: Ventromedial hypothalamus
WAT: White adipose tissue
β3-AR: β3 adrenocepter
